# Exploring a practitioner-athlete relationship and facilitated learning throughout a psychological skills training program

**DOI:** 10.3389/fpsyg.2024.1354129

**Published:** 2024-03-26

**Authors:** Xiao Zhang, Morgan Rogers, Penny Werthner

**Affiliations:** Faculty of Kinesiology, University of Calgary, Calgary, AB, Canada

**Keywords:** sport psychology practitioner-athlete working relationship, learning processes, psychological skills training program, career transition, instrumental case study

## Abstract

Psychological skills training (PST) programs have been consistently reported as an important part of preparation for optimal performance in high performance sport. However, there is much less research about the quality and characteristics of the working relationship between a sport psychology practitioner (SPP) and an athlete and, importantly, how that relationship facilitates learning. Therefore, the purpose of the present paper was to explore the working relationship between a SPP and a volleyball player and how that working relationship facilitated the learning processes utilized by this player, as she prepared for the demands of her sport and life. An instrumental case study methodology with a qualitative description approach was employed to illustrate different aspects of the evolving relationship and the athlete’s experiences. The results of this case reflect an approach that combined features of both a directive approach in teaching specific psychological skills and a less directive and more collaborative approach, which, in turn, allowed an athlete to begin to learn how to guide their own learning.

## 1 Introduction

Over the last three decades, the field of sport psychology has become much more accepted as an important part of preparation for optimal performance in high performance sport. Psychological skills training (PST) programs have been designed to enable athletes to learn critical skills such as focus, self-talk, setting of effective goals, visualization, competition planning, and debriefing (Jones et al., 2002; Connaughton et al., 2010; Beauchamp et al., 2012; Wang and Zhang, 2015). Development of these skills has also been shown to enhance the psychological well-being and mental health of athletes (Horn et al., 2011; Golby and Wood, 2016; Foster and Chow, 2020; Durand-Bush et al., 2022). In particular, effective goal setting is one of the key strategies of a psychoeducational model which is designed to foster athletes’ life skills through sport and assist athletes in career transition (Danish et al., 1993; Lavallee, 2005; Kendellen and Camiré, 2017).

Weinberg and Gould (2015) define a PST program as a “systematic and consistent practice of mental or psychological skills for the purpose of enhancing performance, increasing enjoyment or achieving greater sport and physical activity self-satisfaction” (p. 248). The premise of PST programs is that optimal performance occurs when athletes are able to regulate their internal functioning such as cognition, emotions, and sensations. When negative thoughts or emotions occur, athletic performance is more often than not interrupted (Hardy et al., 1996). Thus, the goal of a PST program is to develop an athlete’s capacity to monitor and manage their thoughts, feelings, and behaviors. It is also suggested that a PST program needs to be sequenced to meet the various needs of different athletes in different sports and periodized and integrated with the physiological preparation to ensure the greatest benefit (Balague, 2000; Holliday et al., 2008; Blumenstein and Orbach, 2020). Specifically, psychological skills such as visualization, self-talk, and recovery, have been examined as an effective way to enhance performance and reduce physical and mental fatigue within the sport of volleyball, a sport characterized by high intensity and short duration with a high incidence of injury (Shoenfelt and Griffith, 2008; Fortes et al., 2020; Xu et al., 2020; Coimbra et al., 2021).

Often, a PST program involves a multimodal format integrating the psychological skills systematically to enable an athlete to reduce negative thoughts, control potentially harmful distractions, and increase self-awareness and confidence (Hardy et al., 1996). Importantly, these programs have been frequently employed to not only enhance performance (Thelwell and Maynard, 2003; Sheard and Golby, 2006; Vealey, 2007; Birrer and Morgan, 2010; Beauchamp et al., 2012; Wang and Zhang, 2015), but also enhance an athlete’s positive psychological profile (Sheard and Golby, 2006; Golby and Wood, 2016).

Understandably, the majority of the research in the field has been focused on the interventions utilized to develop these psychological skills (Barker et al., 2020). Much less research has been dedicated to exploring the quality and characteristics of the working relationship between the sport psychology practitioners (SPPs) and clients (Tod and Andersen, 2012). Research in the field of counseling psychology, highlighting the critical importance of the practitioner-client working relationship, can help us understand the value in developing such relationships (Duncan et al., 2010), and scholars and SPPs in the field of sport psychology have argued that the SPP-athlete relationship is similar (Petitpas et al., 1999; Tod and Andersen, 2012).

Practitioner opinion pieces highlight the importance of trust, rapport, and caring as vital for developing the SPP-athlete relationship (Fifer et al., 2008; Werthner and Coleman, 2009). Research exploring consultant views on such relationships, conducted by Sharp et al. (2015) as well as Arnold and Sarkar (2015), suggest knowledge and expertise, ethical behavior, rapport, respect, trust, and being available for support are hallmarks of an effective SPP-athlete relationship.

Tod et al. (2023) explored SPPs’ narratives of client-led approaches to working with athletes and highlighted that such an approach reflects, at least in part, adopting a person-centered therapy approach (Rogers, 1959). The work of Rogers (1961, 1963) emphasized building a relationship based on empathy, respect, and authenticity and a strong belief that clients are capable of determining their own behavior, dependent upon a number of conditions. Nevertheless, in the article by Tod et al. (2023), the authors acknowledge that SPPs in sport psychology may use both a client-led approach, which is non-directive and encourages the athlete to play an active role, and a SPP-led, more directive role, determining the specific psychological skills to be learned. This combination of a non-directive stance with a directive framework, according to the authors, makes sense given the setting of high-performance sport.

In turning to consider how a SPP-athlete relationship might also facilitate the learning of specific psychological skills, it is useful to reflect briefly on the process of learning. One theory of learning that looks at learning from a constructivist view of learning, focuses on how an individual makes sense of their world, and suggests two views of learning to help us understand the actual process: the “building a brick wall” and “the network” (Moon, 2004, p. 16). The metaphor of building a brick wall implies that there is an instructor who provides the information that builds one’s knowledge. It is assumed that the instructor knows best how the “bricks” of knowledge will fit together and suggests, without instruction, there is no learning. Her second metaphor is described as “a vast but flexible network of ideas and feelings with groups of more tightly associated linked ideas/feelings” (Moon, 2004, p. 16). In this view, learning takes place in many different ways and an instructor may or may not be present. Learning from this perspective is seen as a life-long process, taking into account what an individual already may know, at any point in time, and how new learning may, or may not, be incorporated into what Moon (2004) calls an individual’s “cognitive structure” (p. 17). In other words, one can learn in a course, with an instructor facilitating learning and one can learn by reflecting on current knowledge and develop a deeper understanding of a particular idea or topic. This theory and both views of learning can help us frame an understanding of how an athlete may learn psychological skills from the instruction of a SPP and, at the same time, learn how to reflect, ask questions, try out different strategies and incorporate the developing expertise into what they are experiencing in their on-going sporting experience.

Therefore, the purpose of the present paper was to explore the working relationship between a SPP and a volleyball player and how that working relationship facilitated the learning processes utilized by this player as she prepared psychologically for the demands of her sport and her life.

## 2 Methods

### 2.1 Methodology

The philosophical paradigm of the present research is situated in constructivism suggesting the presence of multiple, subjective realities (Sparkes and Smith, 2014). Individuals experience and make meaning of these multiple realities in different ways and constructivist research relies heavily on research participant voices and the contexts and cultures that give rise to their experiences (Creswell and Poth, 2013). Qualitative description is an approach that provides an in-depth summary of a participant’s experience in easily accessible language (Sandelowski, 2000; Bradshaw et al., 2017; Doyle et al., 2020).

Case studies are specific and bounded and allow us to develop an in-depth understanding of a particular event or issue (Creswell and Poth, 2013; Hodge and Sharp, 2017). Instrumental case studies focus on a specific topic, where a case is selected for its ability to illustrate the chosen issue (Creswell and Poth, 2013; Hodge and Sharp, 2017). The present research uses an instrumental case study to create a rich illustration of the development of a working relationship between a SPP and an athlete as well as how that working relationship facilitated the learning processes utilized by the player.

### 2.2 Procedure

Ethical approval was obtained from the researchers’ university ethics board for the design and implementation of an 18-month PST program as well as the present research. The first author joined a Chinese provincial professional women’s volleyball team as a SPP and researcher.

#### 2.2.1 The setting of the study

The present study was conducted with one female volleyball player who played on three different teams over the course of this 18-month intervention (two Chinese provincial professional volleyball teams and a Chinese university women’s volleyball team). As part of a larger study on PST with two Chinese volleyball teams based in Beijing, China, an introductory meeting and a series of team sessions were held from April to July 2021. Many of the sessions were held in person as the SPP was embedded in the team setting. However, several individual consultations and a third interview were conducted online via WeChat (a multi-purpose social media and messaging app used as both a work app and communication app in mainland China, allowing users to send messages, make voice and video calls, share photos and videos etc.) due to the on-going presence of COVID-19. All team sessions were formal and lasted approximately 1 h and the individual sessions ranged from 45 min to 1 h.

At the time of the study, one of the researchers (first author) was a PhD candidate under the supervision of a professor at a Canadian university who also works in the field of sport psychology (third author). The first author was born in China, earned her undergraduate degree in China, and then completed a master’s degree in the United States. The first author’s ability to speak mandarin Chinese and understand Chinese culture, along with her education, provided her with an insider-outsider viewpoint and an ability to work effectively with Chinese athletes (Berger, 2015).

#### 2.2.2 Participant

During the team sessions, one player approached the SPP and asked if she could meet individually with the SPP. As a result, the present study is an exploration of the work with that individual athlete over a period of 18 months. This athlete started playing volleyball at a sport school at the age of 14. Two years later, at the age of 16, she began playing professional volleyball at the national and international level. At the age of 19, she entered university as a professional player. At the age of 20, she decided to leave professional volleyball but continued to play the sport as a student-athlete.

#### 2.2.3 Data collection

Once the intervention was agreed upon, the athlete signed a consent form, and an in-depth, semi-structured interview was conducted by the SPP. The purpose of the first interview was to ask about the athlete’s experiences in sport and her current understanding of various psychological skills (e.g., Tell me about your sport performance to date. Tell me what you know about psychological skills and how you currently manage the stress of competition). After this initial interview, the athlete participated in an 18-month PST program consisting of four team sessions with teammates and regular individual meetings with the SPP, while training and competing on two provincial level professional women’s volleyball teams and subsequently on a university team.

Two subsequent interviews were conducted to understand how the athlete was learning the psychological skills and utilizing those skills in training and competition, as well as in other parts of her life (e.g., Please tell me what skills, if any, influenced your performance in competition, in school, and in your life in general? Tell me about what might have been new for you as we worked on preparing for training, competition, and your studies? What skills, if any, might help you as you consider transitioning out of high-performance sport?) (See Table 1 for timeline.)

**Table 1:** Timeframe of the PST program.

Date	Sessions			Place
April–July 2021	Team sessions	Introduction to sport psychology and psychological skills training	4 sessions	Beijing, China
		Goal setting, focus, self-talk, visualization etc.		
		Debriefing and competition plans		
		Team cohesion		
	1st interview			
	Individual consultations		12 times/month	Beijing, China and Online via WeChat
August–October 2021	Individual consultations		2 times/month (The athlete was away at home or at school, playing as a student-athlete.)	Online via WeChat
November–December 2021	Individual consultations		2 times/week	Jiangmen, China and Online via WeChat
January–September 2022	Individual consultations		2 times/month (Due to COVID-19, the athlete spent most of time at home.)	Beijing, China and Online via WeChat
	2nd interview		In person	
	3rd interview		Online via WeChat	

The three semi-structured interviews were conducted in mandarin Chinese, audio-recorded, and transcribed verbatim by the first author in mandarin Chinese. All identifying information was removed from the transcripts. The first author sent the verbatim transcripts in mandarin Chinese to the participant and no changes were suggested. The transcripts were then translated into English, resulting in 26 single-spaced pages. As well, entries from the first author’s reflexivity journal are included in the results.

#### 2.2.4 Data analysis

Doyle et al. (2020) suggest analysis in a qualitative descriptive study should be inductive, without transforming data beyond recognition, with the goal of providing a comprehensive summary of participant experiences that remain close to their original account. Therefore, data were analyzed using a hierarchical content analysis which examines what has been said by a participant. Patterns in the data are categorized and described in a way that allows general knowledge about a topic to be developed (Sparkes and Smith, 2014; Bradshaw et al., 2017). Sparkes and Smith (2014) outline the following procedure for a hierarchical content analysis: immersion in the data, identifying themes, connecting themes, reviewing themes, further refining themes with another investigator, and presenting results. The transcripts were thoroughly reviewed several times to familiarize the authors with the data set. Following this, an initial set of themes were developed, to ensure a level of interpretation while remaining “data-near” (Sandelowski, 2010, p. 78), discussed extensively between the first and third authors, and refined.

#### 2.2.5 Rigor

Given that Sandelowski (2000) notes that data cannot exist in a theoretical vacuum and qualitative descriptive studies will still be influenced by the researchers’ lived experience, the first author kept a reflexive journal throughout the 18-month period, documenting her thoughts, impressions, and questions that emerged from implementation of the team sessions and individual consultations, and the primary author’s PhD supervisor (third author) acted as a critical friend throughout the research process (Smith and McGannon, 2018). There were bi-weekly conversations between the first author and her supervisor, discussing what was working, what the SPP was struggling with, and what questions she had for the next series of individual sessions.

## 3 Results

As noted by Sandelowski (2000), qualitative descriptive data are represented in a way that best “fits the data” (339) and, as a result, for the purpose of the present study, the findings are presented chronologically to illustrate how Yolanda (a pseudonym) experienced the PST program, how she learned throughout the 18-month intervention, and how the working relationship between Yolanda and the SPP evolved over the 18-month time period.

### 3.1 In the beginning

In April and May of 2021, the SPP provided four team sessions on the development of psychological skills to one of the two professional teams. Yolanda was part of this team. Two times per week, the coaches also asked the SPP to choose a psychological skill to speak about with the players prior to the training session. As well, during the weekly team meeting, the SPP addressed specific skills based on what she observed in the training.

In one of the early sessions, the SPP asked each of the players to write down what they thought they might need to work on to be better prepared psychologically for both training and games. Yolanda attended all four team sessions but did not initially interact with the SPP and wrote very little on paper. Yolanda spoke of the first few team sessions and how she was reluctant to share what she thought:

*When you came to the team at that time, I was not familiar with you and I did not listen to your presentation very carefully, because there were often many people who came to give lectures to the whole team that were not helpful to me. So, I took this lecture time as relaxation. I did not dare write the truth when I answered your questions because I was afraid that the coach would read it, and that might not be good.*

This early reflection from the athlete speaks, in part, to the time it takes to build toward a productive working relationship. Certainly, living in the training center and regularly attending practices, video analysis sessions, and games, to both observe the context and begin to interact with the athletes in general and Yolanda specifically, enabled the SPP to begin to build a connection with Yolanda. The SPP also spoke with her supervisor (third author) about how to develop a relationship with Yolanda, and reflected in her journal:

*The main task of the first few weeks is for me to learn more about volleyball, to let the team know my background and my role in the team, and to start slowly building rapport with Yolanda by observing her practices and listening to her. At this point I was still providing team sessions to the whole team and not yet conducting individual sessions with her.*

### 3.2 The individual sessions

In June 2021, following the conclusion of the team sessions, Yolanda approached the SPP to ask if they could continue to work together individually and spoke of a number of concerns she was facing. She was worried about her ability to play well consistently, she was worried about her teammates and coaches knowing she was meeting individually with the SPP, and she was also concerned about them knowing she was considering transitioning from being a professional player to a student-athlete. However, Yolanda noted the SPP’s approach of maintaining confidentiality strengthened their burgeoning relationship.

*Gradually, I realized that you would not tell what I told you to the coach, like the first time you asked us to answer questions on training, you did not report to the coach, which made me trust you a lot. And in our daily conversations, you always ask what I need, even when it’s unrelated to training.*

To better understand Yolanda’s decision to make a sport career transition at the age of 20, as well as continue to develop rapport, the SPP asked Yolanda about her experiences in the sport, including what motivated her and what she enjoyed about playing volleyball. Yolanda shared that her primary motivation to play volleyball was to “get a National Elite Athlete certificate to enter a national level university as a student-athlete.” Yolanda had already been considering transitioning from professional volleyball to being a student-athlete for several months when she began working with the SPP.

Because Yolanda was planning to transition from professional sport to university sport, she initially sought out the SPP for support on her academics and returning to full-time university, rather than focusing on skills for training and competing. As Yolanda and the SPP began to discuss how Yolanda might return to school as a student-athlete and transition from the professional team to a university level team, the SPP took time to learn more about the university sport setting. The SPP noted in her journal that Yolanda would need to balance studying with training, so she planned to work with Yolanda on goal setting and debriefing, to better help Yolanda adapt to the stresses of university life as a student-athlete. The SPP also suggested that working together on the various psychological skills while continuing to train with the professional team could benefit her as a student-athlete when she transitioned to the university team.

Therefore, Yolanda and the SPP started working on specific psychological skills to manage the stress of training and competition. Working on these skills was new to Yolanda, as she shared that she had taken a sport psychology course in university, but the course was “mostly about theories rather than the applied knowledge helping my performance.” As well, Yolanda mentioned she “could not remember what you (the SPP) talked about in the team sessions very well.” Individual sessions involved working on how to focus effectively in practice and games, how to develop a clear understanding of her role and position on the team, how to practice the skill of visualization through “seeing” and “feeling” different plays and possible reactions to various scenarios, learning how to set effective daily and longer term goals, and spending time reflecting on each training session to be better prepared for the next day of training.

*In the past I was passive (in training) and did not want to practice. I think I can now talk about my own understanding of volleyball. It’s all after you came to the team during this period. I never had this kind of help before. It makes no sense chatting with friends or parents. They have never been in this competitive environment. The best they can do is just encourage me to be positive.*

Yolanda also worked with the SPP on her communication skills and being more proactive in training and interactions with her coach. Despite her challenges with English, Yolanda was also keen to participate in discussions between the SPP and the European strength and conditioning coach, relying on the SPP’s assistance for translation. The following quote from Yolanda illustrates how she began to take an active role in her training.

*Training with him (the strength and conditioning coach) was quite different from other Chinese coaches. He taught us how to do each movement clearly, told us how the muscles work and explained why he asked us to do so, rather than just doing squats with heavy weights. Training with him is not easy, but I want to put all my effort in and ask him questions about how to strength train to improve my spiking in competition. You (the SPP) were helping with translation in the training sessions with us, which made me feel more comfortable to talk with him. Also, I could learn certain English words in volleyball through your translation and make notes in my daily journal.*

From June to August 2021, Yolanda kept a daily reflective journal, which she shared with the SPP on an almost daily basis. In addition to seeking out the SPP in person to share her daily reflections, Yolanda and the SPP occasionally spent time together working together on her schoolwork. The SPP noted in her own journal that, in addition to individual sessions working on the psychological skills, discussing Yolanda’s daily journal notes helped to develop Yolanda’s ability to articulate her thoughts and emotions as well as apply what she was learning to both her training and studies.

*Although Yolanda cannot communicate very well with the strength and conditioning coach due to language barriers, she wants to learn more about the research on strength training in women’s volleyball. With my help in translation, she was able to start applying what she learned in her own training. Yolanda reflected on these conversations in her daily journal and discussed with me. Also, Yolanda enjoyed studying together in the evenings. I would work on my own work, and we would sometimes discuss what she was learning in school. I helped her when I could.*

In late August 2021, after many conversations with the SPP regarding what life might look like after professional sport, Yolanda decided, with the support of her parents, to go back to school and yet still play volleyball as a student-athlete. At this point, Yolanda still wanted to work with the SPP.

During this time, it became clear to the SPP that Yolanda was becoming eager to learn more about both her training and her future. She was curious and wanted to learn from the SPP how to “think about” what she was working on in both practice and school. Yolanda wanted to understand how she could learn from each game, how she could better prepare for both training and exams, and how she could balance study and training.

*My life used to be simple while living in the professional team. There were three places that I would go every day: dorm, canteen, and court. I did not need to care about anything except training. It was just high intensity and a large quantity of training starting from a very young age on the professional team. Although I do not enjoy playing volleyball now, I am still working hard on the court… when I am training and competing on court, I am still very serious… If I have classes after, I will go to class. If no classes, I would continue to study in the classroom until the building is closed. Then I go back to the dorm and go to bed directly. I feel very tired, but very satisfied.*

As Yolanda’s interest in time management and dealing with multiple tasks grew, the relationship and work between the SPP and Yolanda evolved. Over time, the SPP began helping Yolanda adapt to university life alongside enhancing her training and performance. As well, at Yolanda’s request, the SPP helped Yolanda maintain a number of processes and create a few new ones - the development of a plan for each day and then a debrief process at the end of each day of training and school to reflect on how it all went. As part of these changes, Yolanda reflected on the importance of writing.

*I think writing down my thoughts is really better than just thinking. I thought I could remember everything by thinking rather than writing. But I found that some of my training information was missing. I think when I write it down, I have all the details clearly articulated. I think the main purpose of a writing journal is to help me sort out what I practiced today and my performance, what I did not do well, what I should and could do, and what I need to improve for the next training session. And along with writing at the end of training, I think the process of thinking before training may be even more useful for me.*

Similarly, Yolanda created a plan and reflected on it for her life in school and when she had the opportunity to help with team administrative work during the China University Volleyball League.

*For both training and school, I need to take care of myself and finish other tasks the coach or teachers give to me. During the college competition, I worked for the team as the team manager. I had no experience dealing with these administrative issues, but you suggested to me to write it all down, just like what I did before each competition. So I wrote it down on my phone every night. If I did not know how to do it, I would ask for help in advance, and it was always much better the next day.*

These journal reflections, and notes on Yolanda’s phone, often became the basis for discussion in the individual sessions. Whether in person or online, this process enabled Yolanda to think deeply, increasing her self-awareness, and creating a sense of personal agency as her life progressed.

Setting goals and creating a clear purpose for oneself in both sport and life was one of the critical skills introduced to Yolanda in the early team sessions and something she worked on and talked about throughout her individual sessions. As the sessions progressed, Yolanda also began to understand the value of setting small goals for training sessions, school, and her future. She also spoke about learning to use goals as a way to both focus during practice and focus on steps she needed to take to becoming a university student.

*My goal is to get a postgraduate recommendation. Winning the championship for my university in National Sport College Volleyball League is the most likely way for me to get that recommendation. But I also need to study hard to be qualified, that is, to pass CET-4 (College English Test, Level 4), and my grades ranking need to be at or near the top.*

In November 2021, Yolanda was selected to play at a national tournament. After playing as a student-athlete for several months, Yolanda was not confident in her ability to compete again in a high intensity professional competition. This situation also reminded Yolanda of the injuries she had experienced over the course of her career, and as a result, she struggled with her confidence in returning to play as a professional volleyball player.

*In 2020, I sprained my ankle when I was blocking on the right forward during training and I have not recovered very well from that. Now, I am very afraid to be injured again. I do not even have a picture in mind that I ever did this movement well. I thought I could not play well anymore.*

To slowly rebuild her confidence and enable her to become ready to return to playing professional volleyball in the tournament, the SPP and Yolanda continued to work together on the skill of visualizing different plays, on differentiating between effective and ineffective thinking, and practicing self-talk that would help rebuild her confidence.

*I think visualization is very helpful in making me think deeply and correctly every day. For example, for the right forward blocking, I watch my favorite player’s blocking video and then visualize how I do that movement on court. After watching, I have it in mind and then I can visualize how I do it and keep it in mind every day. It helps so much.*

As the individual sessions progressed during the professional tournament in November and December 2021, Yolanda worked on developing a clear plan for each day of training, and felt she began to truly learn how to take control of what she was thinking and how to manage her emotions when things did not go well in training and competitions.

*At least, through the meetings with you, I could calm down and figure out what I could do for the next game or competition. No matter if the team wins or not, I just focused on myself, what I could do. A good spike needs working with the setter, but for blocking, I could do that by myself. And I focused on this competition as a chance to improve myself. After each game, I debriefed with you --about what you asked me to think about. I know a lot about high-level players and now have a clearer understanding of my own strengths and shortcomings. I thought what you taught me is to debrief after training or competition, and to make a plan before training or competition, to help me think clearly, and focus on what I can do, rather than to only care about the results or evaluations of others.*

### 3.3 Concluding

In January 2022, Yolanda had just finished participating in a national competition as a professional player. She expressed excitement about embarking on a new chapter in her life but also admitted feeling nervous and unsure about being a capable university student and student-athlete. Transitioning from being a professional athlete to a student-athlete with the lower intensity of training made training relatively easy for Yolanda. However, having spent a number of years training as a professional player with limited academic classes, Yolanda lacked confidence in her studying ability.

*Training with the university team is easier and less time-consuming for me compared to my experience with the professional team. But it is harder for me to get a good GPA than a good performance on court. I know I have to work hard and put a great deal of effort into studying…but is hard for me to focus on studying. I have been training in a professional sport team without getting a formal education for such a long time. I know it will be difficult for me to catch up with normal students. But at least I now know how to focus.*

Due to the pandemic in China in early 2022, Yolanda explained she spent six months at home with her family, taking online courses and training. Compared to the previous two months of attending school on campus, Yolanda shifted her emphasis to enhancing her learning abilities and thinking about her future. The conversations between Yolanda and the SPP continued, in part, to be on learning, and on Yolanda’s vision for her future life.

*At present, my plan is to complete my master’s degree. The main thing is that I do not know what I can do in the future. Being a coach or a teacher may not be very easy for me, but I will try my best to pass the exam to get a teaching qualification in the primary and secondary school. It could be a backup. At least it could let me be independent in the future.*

While staying at home from January to early August 2022, the SPP helped Yolanda with the preparation for her exams and with developing a routine. Along with schoolwork, the SPP also helped Yolanda work on her preparation for English exams and a teaching certificate. With that help, Yolanda gradually began developing learning habits which, she said, “make me very comfortable.” Through these conversations and sessions, Yolanda applied the skills she used in sport to her schoolwork and came to understand that setting small goals for herself, on a daily basis, enabled an effective focus for studying, and brought her happiness and a sense of accomplishment.

*Yes, my long-term goal is a postgraduate recommendation. To split it up and set some small goals for myself, which can be achieved at each stage, just like the psychological feelings of eating melon seeds. Why do people love eating melon seeds so much? Because we can get happiness in the moment, we do not have to wait so long. And mapping out those goals is such a good idea.*

In mid-August 2022, post-pandemic and lockdown, Yolanda returned to begin a new semester in person and adapted well to university, enjoying taking classes where she found the instructors were “more like friends,” whereas previously she felt “super scared and nervous when talking with the instructors and the staff.”

*I think taking class is quite interesting. The teachers are humorous. When I was on the professional team, I had a passive attitude toward everything. The coaches always pushed me. I cannot say how much knowledge I got during this period. Now I am back on campus, I find that the excellent teachers never push students in class and yet we are eager to learn from them.*

As part of supporting Yolanda’s transition to full-time school, the SPP occasionally shared some of her own experiences of attending university in China and then her graduate education abroad, inspiring Yolanda to broaden her vision.

*What you talked to me about is not only helpful in sport but also in life, such as your experiences over the years. I have neither seen nor considered many things that you talked with me. I so like to talk with you and wish I could go abroad to know the world. I am willing to listen to your advice and try my best.*

In summary, the working relationship between the SPP and Yolanda developed substantially over the 18 months. In the beginning the focus of work was on learning and practicing a variety of psychological skills for both training sessions and game situations. As the trust and rapport progressed between the two individuals, the conversations and work together expanded to include discussions of transition from professional sport and to becoming a fulltime university student and student-athlete.

## 4 Discussion

In this case study, we explored the working relationship between a SPP in sport psychology and an athlete, how that relationship evolved over an 18-month period, and how it facilitated the learning processes of the athlete.

In beginning, this athlete was primarily motivated by an opportunity to attend a good university and playing volleyball was her entry point. As work with the SPP progressed, she began to enjoy her sporting experience and to understand how developing psychological skills and learning about herself could help her performance in both training and competition.

Certainly, it took time for the SPP to build rapport with this athlete. This was accomplished, in part, by the SPP living in the training environment, consistently attending practices and games, and engaging with the athlete on a regular basis over more than a year. In the beginning, the sessions were focused on teaching the critical skills needed for sport performance and then slowly evolved into conversations and work that helped the athlete learn how to reflect on the demands of her sport and her increasing interest in future education and a career outside of sport.

In addition to rapport, trust was built between the SPP and athlete by the skills the SPP brought to the setting and her perspective – a keen desire to understand the athlete, a willingness to listen to her concerns and questions, an understanding of the importance of confidentiality, and an ability to create an environment of empathetic understanding of the athlete’s world. The latter points to what Rogers (1961), many years ago, intended by the notion of person-centered therapy. These results also support the more recent research of Tod et al. (2022) that notes a number of critical practices of effective SPPs, such as building rapport, developing a relationship based on openness, and operating well in the athlete’s context. It also reminds us of the much earlier work of Orlick and Partington (1988) which emphasized the importance, for SPPs, of active listening, presence, and empathy.

As the working relationship developed, the SPP was willing to allow the athlete to lead in the sessions and direct what was discussed and worked on. This evolution in the relationship speaks to what is perhaps required for those working in a high-performance sport environment as SPPs. In this study, it is clear that a skilled SPP needs to be able to understand the benefits of combining both a directive approach in teaching specific psychological skills and a less directive and more collaborative approach, allowing an athlete to begin to learn how to guide their own learning. Nevertheless, determining when and how to combine these two approaches would be dependent on a number of factors, such as the maturity and experience of each athlete.

In reflecting on the learning processes illustrated in this case study, we are able to see how this particular athlete engaged with and learned the psychological skills over a competitive season, how she matured, and what she found personally meaningful. As Moon (2004) suggested, “‘meaningfulness’ is an individual judgment” (p. 18). In Moon’s theory of learning, meaning is created by the learner both through what she calls “material of learning” (Moon, 2004, p. 19), which in this case was the PST program and learning how to reflect on both the material being presented and her own experiences. Moon (2004) suggests that reflection is “often a process of re-organizing knowledge and emotional orientations in order to achieve future insights” (p. 82). In the case illustrated in the present research, this process was enabled by each interaction and conversation with the SPP in each of the sessions through teaching, listening, asking questions, and listening again. This athlete began to move from an individual who reflected very little on her training, to someone who began to understand, appreciate, and act on a regular process of planning, executing as best she could, in training and games, and debriefing and reflecting on her training, and eventually her life more broadly.

Finally, what occurred over time in this working relationship was a discussion about transitioning out of sport and it is well known that this process is rarely easy for athletes who have devoted much of their early life to sport (Werthner and Orlick, 1986; Li et al., 2022). Yolanda was initially unaware of how she might create a life outside of sport due, at least in part, to her limited experience in education and managing her own life. As work with the SPP progressed, Yolanda was able to think more deeply about her life, learn to enjoy her university life, and commit to pursuing her education. This work reminds us that the field of sport psychology is not just about sport performance but also about helping athletes develop a healthy life in and after sport (Zhang et al., 2013, 2019).

## 5 Limitations and future directions

There are three potential limitations of this research. A limitation of the qualitative descriptive design is its lack of a strong theoretical framework and its primarily descriptive nature, which may result in a potential lack of nuance in the findings (Neergaard et al., 2009). However, it is argued the descriptive design aligns well with the purpose of the present case study, where the aim was to explore the working relationship between a SPP and an athlete and how it may have facilitated the learning experiences of a Chinese volleyball player throughout a PST program. A second potential limitation is that the interviews and consulting work were conducted in mandarin Chinese and then translated into English. There is always a concern that subtleties might be missed in any translation and as a result, a reverse translation, also known as “back translation” was also conducted, which involves, in this case, translating the English text back into mandarin Chinese. However, no issues were found. Finally, given the first author conducted both the PST program and the interviews, a third potential limitation is the possibility that the athlete might have been inclined to emphasize the positive aspects of her experience throughout the working relationship.

There are a number of studies exploring Chinese athletes’ experiences of PST (Wang and Zhang, 2015), mental health (Si et al., 2021), and career transition and life skill development (Zhang et al., 2017; Li et al., 2022; Zhu et al., 2022). The present research illustrates how a working relationship was developed and contributed to developing the learning processes of an athlete, within the context of the Chinese sport system. It is suggested that future research could explore how SPPs in different cultures or contexts can effectively facilitate the learning process for both sport performance and transition out of sport.

## 6 Conclusion

In conclusion, this case study illustrates a working relationship between a SPP and an athlete and how that relationship, in turn, facilitated the learning processes utilized by this player as she prepared psychologically for the demands of her sport and her life. The case also helps us understand that an athlete can play an integral role in her own learning. The SPP certainly created a PST program, which in Moon’s generic view of learning could be considered the “bricks of knowledge” (2004, p. 16) and yet, with the SPP’s facilitative and empathetic skills, also enabled the athlete to learn how to reflect on and learn from her day-to-day experiences. As a result, her sport skills improved and she was becoming a more mature athlete and individual. The athlete progressed from an individual who reflected very little to an individual who began to appreciate and act on her own understanding of the importance of planning, executing as best she could in training and games, and debriefing and reflecting on her training, and eventually her life more broadly.

## Data availability statement

The raw data supporting the conclusions of this article will be made available by the authors, without undue reservation.

## Ethics statement

The studies involving humans were approved by University of Calgary/ Conjoint Health Research Ethics Board. The studies were conducted in accordance with the local legislation and institutional requirements. The participants provided their written informed consent to participate in this study.

## Author contributions

XZ: Writing – original draft, Conceptualization, Formal analysis, Investigation, Methodology. MR: Methodology, Writing – review & editing. PW: Supervision, Writing – review & editing, Conceptualization, Methodology.
